# Bleomycin-Induced Lung Toxicity in Hodgkin's Lymphoma: Risk Factors in the Positron Emission Tomography Era

**DOI:** 10.7759/cureus.23993

**Published:** 2022-04-09

**Authors:** Selim Jennane, Mounir Ababou, Mariyam El Haddad, Omar Ait Sahel, El Mehdi Mahtat, Hicham El Maaroufi, Abderrahim Doudouh, Kamal Doghmi

**Affiliations:** 1 Hematology Department, Mohammed V Military Hospital, Mohammed V University, Rabat, MAR; 2 Nuclear Medicine Department, Mohammed V Military Hospital, Mohammed V University, Rabat, MAR

**Keywords:** chemotherapy-related toxicity, risk factors, hodgkin lymphona, fluorodeoxyglucose positron emission tomography, bleomycin-induced lung injury

## Abstract

Introduction

Bleomycin is a major antimitotic agent in the first-line treatment for Hodgkin's lymphoma. The main limitation of its use is its pulmonary toxicity. The objectives of this study are to find out the risk factors for the occurrence of bleomycin-induced lung toxicity in patients with Hodgkin's lymphoma and, on the other hand, to determine if positron emission tomography scan is a reliable means of early detection of this toxicity.

Methods

This is a retrospective study conducted in the clinical Hematology Department of Mohammed V Military Instruction Hospital, Rabat, Morocco. All patients with Hodgkin's lymphoma and treated with a bleomycin-based chemotherapy were included. The impact of different clinical and biological factors on the risk of bleomycin-induced lung toxicity occurrence was assessed using univariate and multivariate logistic regression. The benefit of positron emission tomography, usually performed as part of the re-assessment of Hodgkin’s lymphoma after two and four cycles, has been evaluated in the detection of bleomycin-induced lung toxicity.

Results

Among 124 patients included in the study, 18 (14.5%) patients experienced bleomycin-induced lung toxicity. On multivariate analysis, smoking (p = 0.038) and the use of the ABVD regimen (doxorubicin, bleomycin, vinblastine, and dacarbazine) compared to the escalated BEACOPPe regimen (bleomycin, etoposide, doxorubicin, cyclophosphamide, vincristine, procarbazine, and prednisone) (p = 0.018) were statistically significant risk factors.

After two and four courses of therapy, the positron emission tomography was able to predict the occurrence of bleomycin-induced lung toxicity before the appearance of clinical symptoms only in 36.4 % and 12.5% of patients, respectively.

Conclusion

Studies to identify risk factors for the development of bleomycin-induced lung toxicity are crucial to reduce toxicity in the treatment of Hodgkin's lymphoma. However, two- and four-cycle positron emission tomography scans cannot be considered as a reliable means of early detection of this toxicity.

## Introduction

Bleomycin is an antimitotic agent used mostly in germ cell tumors and Hodgkin's lymphoma (HL). The main limitation to its use is the risk of interstitial pulmonary fibrosis [1,2]. This is an unpredictable and potentially fatal complication that occurs in one out of five HL patients treated with bleomycin-containing chemotherapy [3]. The identification of risk factors (RFs) for its occurrence is therefore essential in order to limit its use in patients at risk. The objectives of this study are to find out the RFs for the occurrence of bleomycin-induced lung toxicity (BLT) in patients with HL, and on the other hand, to determine if positron emission tomography (PET) scan is a reliable means of early detection of this toxicity.

## Materials and methods

This is a retrospective study conducted in the Clinical Hematology Department of the Mohammed V Military Training Hospital, Rabat, Morocco. All patients with HL treated with a bleomycin-containing regimen were included. The inclusion period covered the period from January 2007 to August 2020.

Characteristic of the studied sample in comparison with the Moroccan population (bias in the recruitment)

The Clinical Hematology Department of the Mohammed V Military Training Hospital takes care of military personnel and their dependents aged 16 years and above. Therefore, there is a slight male predominance in the recruited patients compared to the general population.

Therapeutic management

In first-line therapy, patients were treated with two different chemotherapy regimens: ABVD regimen (including doxorubicin, bleomycin, vinblastine, and dacarbazine) and escalated BEACOPPe regimen (including bleomycin, etoposide, doxorubicin, cyclophosphamide, vincristine, procarbazine, and prednisone) [4-6]. The ABVD regimen results in less hematological toxicity but involves a higher dose of bleomycin (10 mg/m^2^ on D1 and D15 corresponding to 20 mg/m^2^ per 28-day cycle). The escalated BEACOPPe regimen is more hematotoxic with less bleomycin (10 mg/m^2^ per 21-day cycle) [4-6]. The choice between these two regimens is discussed on a case-by-case basis during a multidisciplinary consultation meeting (MCM) and depends on the patient's age, comorbidities, and stage of the disease.

In stage I and stage IIA (Ann Arbor staging classification), patients were treated with ABVD regimen with or without involved-field radiation therapy. In stage IIB and disseminated stages (stages III and IV according to Ann Arbor staging classification), elderly patients or patients with comorbidities were treated with the ABVD regimen and young patients without comorbidities were treated with escalated BEACOPPe regimen. Granulocyte colony-stimulating factor (G-CSF) was systematically used in the BEACOPPe regimen from D9 to neutrophil recovery (i.e., a neutrophil count greater than 0.5 G/L). In the ABVD regimen, G-CSF is used on a case-by-case basis depending on weekly monitoring of the neutrophil count.

As part of the extension assessment, a pre-therapeutic PET scan is systematically performed in all patients. Monitoring of treatment response is ensured by performing an intermediate PET scan after two and/or four cycles (depending on the decision of the MCM) and an end-of-treatment PET scan.

Diagnostic criteria for bleomycin-induced lung toxicity

In the absence of a reliable diagnostic tool, BLT is a diagnosis of exclusion that is based on an array of clinical and radiologic arguments and respiratory function testing data (including decline in diffusing capacity of the lungs for carbon monoxide [DLCO]) and exclusion of other infectious, cardiac, post-radiation and drug (other than bleomycin) etiologies [7-9].

Statistical analysis

Comparative statistical analysis of the quantitative variables was performed using Student's t-test and that of the qualitative variables between groups using the chi-square test. The impact of different clinical and biological factors on the risk of BLT occurrence was assessed using univariate and multivariate logistic regression. The confidence interval (CI) was estimated at a degree of 95%. A p-value of <0.05 was accepted as a statistically significant difference. All statistical tests were performed using the IBM SPSS program (IBM Corp., Armonk, NY).

## Results

During the study period, 124 patients with HL and treated with a protocol including bleomycin were included. The median age was 39 years with a range of 16 to 76 years. Males (62.9%) outnumbered females (37.1%) with a male-to-female sex ratio of 1.7.

Among 124 patients with HL, 18 had experienced a bleomycin-induced lung toxicity (BLT+), that being 14.5 %. Five (27.8%) out of 18 BLT+ patients died due to respiratory distress secondary to BLT, that being an overall reported mortality rate of 4% with a sample including 124 patients. Seven of 18 BLT+ patients experienced BLT after four cycles, three after five cycles, and eight after six cycles. The characteristics of patients included in the study are summarized in Table 1.

**Table 1:** Characteristics of the studied population. ECOG, Eastern Cooperative Oncology Group; EORTC, European Organisation for Research and Treatment of Cancer; IPS, International Prognostic Score; ABVD, doxorubicin, bleomycin, vinblastine, and dacarbazine; BEACOPPe, bleomycin, etoposide, doxorubicin, cyclophosphamide, vincristine, procarbazine, and prednisone

	BLT+	BLT-	p-Value
	(n = 18)	(n = 106)	
Median age (range)	48.5 (16-70)	36 (16-76)	0.033
Sex			
Male	12 (66.7%)	66 (62.3%)	0.72
Female	6 (33.3%)	40 (37.7%)	
Smoking, N (%)			
Yes	8 (44.4%)	16 (15.1%)	0.004
No	10 (55.6%)	90 (84.9%)	
Diabetes			
Yes	2 (11.1%)	10 (9.4%)	0.824
No	16 (88.9%)	96 (90.6%)	
Glomerular filtration rate			
Greater than or equal to 90 mL/min	17	102	0.95
Less than 90 mL/min	1	4	
ECOG score			
0	5 (27.8%)	46 (43.4%)	0.344
1	9 (50%)	46 (43.4%)	
2	3 (16.7%)	13 (12.3%)	
3	1 (5.6%)	1 (0.9%)	
General signs			
Present	15 (83.3%)	78 (73.6%)	0.377
Absent	3 (16.7%)	28 (26.4%)	
Stage of disease			
Localized (I-II)	8 (44.4%)	40 (37.7%)	0.589
Disseminated (II-III)	10 (55.6%)	66 (62.3%)	
Prognosis of localized forms (EORTC), N = 48	N = 8	N = 40	0.112
Favorable	0 (0%)	10 (25%)	
Unfavorable	8 (100%)	30 (75%)	
Prognosis of disseminated forms (IPS), N = 76	N = 10	N = 66	0.716
Low risk	0 (0%)	7 (10.6%)	
Intermediate risk	6 (60%)	34 (51.5%)	
High risk	4 (40%)	25 (37.9%)	
Lung involvement			
Yes	0 (0%)	13 (12.3%)	0.114
No	18 (100%)	93 (87.7%)	
Chemotherapy regimen			
ABVD	8 (44.4%)	23 (21.7%)	0.039
BEACOPPe	10 (55.6%)	83 (78.3%)	
Chest radiotherapy			
Yes	5 (27.8%)	21 (19.8%)	0.455
No	13 (72.2%)	85 (80.2%)	
Median cumulative dose of bleomycin received (range)	90 (60-140)	60 (40-160)	0.99
Initial pulmonary SUVmax, median (range)	1 (0.8-1.3)	1.1 (0.8-4.3)	0.99
Four-cycle pulmonary SUVmax, median (range)	4.2 (1-9.4)	1.2 (0.8-4.1)	0.04
Survival rate at two years	72.2%	96.2%	0.003

**Figure 1:**
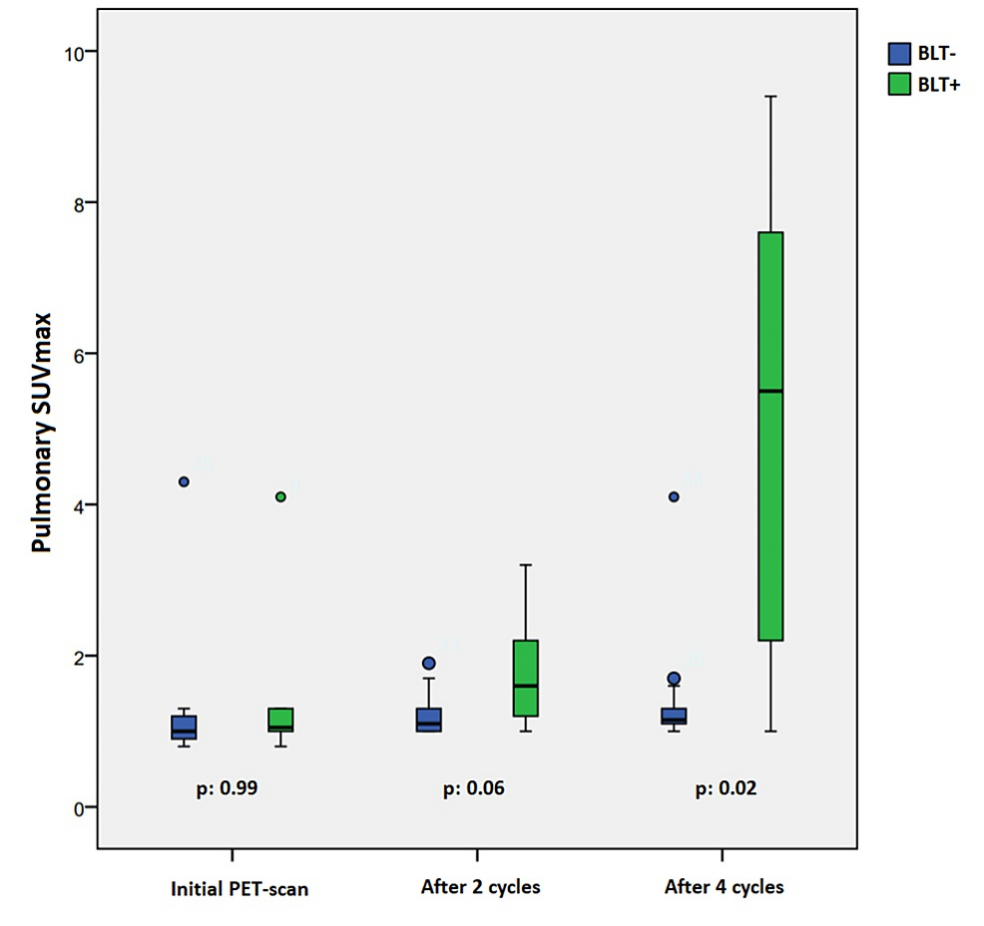
Distribution of pulmonary SUVmax of patients who developed (BLT+) and those who did not (BLT-) on initial PET scan, after two cycles, and after four cycles. BLT, bleomycin-induced lung toxicity

Concerning the benefit of PET scan (Figure 1), there was no statistically significant difference between the initial (p = 0.99) and two-cycle (p = 0.06) pulmonary SUVmax of patients who developed BLT+ and those who did not develop bleomycin-induced lung toxicity (BLT-). The difference between four-cycle pulmonary SUVmax of BLT+ patients and that of BLT- patients was statistically significant (p = 0.04). After two cycles, 11 BLT+ patients underwent a PET scan: four (36.4%) out of 11 patients had pulmonary hypermetabolism before the onset of clinical symptomatology. After four cycles, 16 BLT+ patients underwent a PET scan: nine out of 18 had a pulmonary hypermetabolism (two were asymptomatic, 12.5%, and seven, 43.7%, had already clinical and/or paraclinical symptoms of a BLT). The nine other BLT+ patients did not develop pulmonary hypermetabolism after four cycles, whereas one of these nine patients developed BLT after five cycles and eight after six cycles. Among BLT- patients who had never experienced BLT (n = 106), 85 underwent a PET scan, and four of them were found with diffuse pulmonary hypermetabolism after four cycles. Bleomycin was therefore stopped and the hypermetabolism disappeared at the end of treatment. Clinical examination, radiological data, and spirometric data were normal in these four patients.

Regarding the RFs for the occurrence of BLT (Table 2), variables found to be statistically significant by a univariate analysis were age (p = 0.016), smoking (p = 0.006), and use of ABVD regimen (versus BEACOPPe regimen), with p = 0.03. Based on multivariate analysis, only smoking (p = 0.038) and use of ABVD regimen (p = 0.018) were statistically significant RFs. Sex, disease stage, prognostic score of both the European Organisation for Research and Treatment of Cancer score (EORTC) [10] for localized stages and the International Prognostic Score (IPS) [11] score for disseminated stages, renal function, presence of general signs, and initial HL lung involvement were not statistically significant RFs. The survival rate at two years for patients with BLT (72.2%) was statistically and significantly (p = 0.003) lower than that for other patients (96.2%).

**Table 2:** Risk factors for the occurrence of bleomycin-induced lung toxicity (univariate and multivariate analyses). ECOG, Eastern Cooperative Oncology Group; EORTC, European Organisation for Research and Treatment of Cancer; IPS, International Prognostic Score; ABVD, doxorubicin, bleomycin, vinblastine, and dacarbazine; BEACOPPe, bleomycin, etoposide, doxorubicin, cyclophosphamide, vincristine, procarbazine, and prednisone

	Univariate analysis			Multivariate analysis		
	OR	95% CI	p-Value	OR	95% CI	p-Value
Age	1.04	1.01-1.08	0.016	1.03	0.99-1.06	0.17
Sex	1.21	0.42-3.48	0.72			
Smoking	4.5	1.54-13.13	0.006	3.57	1.07-11.85	0.038
ECOG score	1.66	0.87-3.17	0.12	4.1	0.8-20.95	0.09
General signs	1.79	0.48-6.67	0.38			
kidney failure	NA	NA	0.99			
Ann Arbor stage: localized (I-II) disseminated (III-IV)	0.76	0.28-2.08	0.59			
Prognosis of localized forms (EORTC score), N = 48	NA	NA	0.99			
Prognosis of disseminated forms (IPS score), N = 76	1.46	0.51-4.21	0.48			
Lung involvement	NA	NA	0.99			
Chemotherapy regimen: ABVD vs. BEACOPPe	0.32	0.12-0.89	0.03	0.22	0.06-0.77	0.018
Chest radiotherapy	1.54	0.49-4.79	0.46			

## Discussion

The use of chemotherapy regimens including bleomycin (ABVD and BEACOPPe) has resulted in a relatively high cure rate for patients with HL [3]. Currently, the goal of HL treatment is not only to cure patients but also to limit the toxicity of treatment in the short and long terms [12,13]. BLT is a frequent adverse event, affecting one in five patients (with a range of 9% to 40%) with a mortality rate of about 10% [3]. In addition to its impact on survival, bleomycin may lead to permanent impairment of respiratory function in cured patients [14]. According to the RATHL randomized study, the withdrawal of bleomycin after two ABVD cycles in patients with complete metabolic remission on PET scan does not impact survival and response rates [15]. Other retrospective studies suggest that omission of bleomycin may even be beneficial in elderly patients (>60 years old) regardless of disease stage and response after two cycles of chemotherapy [16,17].

Several teams have been interested in identifying RFs related to the occurrence of BLT. In most of the studies, the median age was higher in the group of patients who developed BLT compared to those who did not develop BLT. Age was also an independent RF for the occurrence of BLT [1,2,18]. However, one study did not show an impact of age, but it was conducted on a young population aged between 26 and 40 years old [19].

In our study, we found a statistically significant difference (p = 0.033) between the median age of patients who experienced BLT+ (48.5 years) and that of BLT- patients who did not (36 years). In univariate analysis, age was a statistically significant RF, but in multivariate analysis, age was not statistically significant.

This result can be explained by the interaction of other RFs that are more frequent in the elderly, such as smoking, use of ABVD regimen (i.e., a higher cumulative dose of bleomycin than in the BEACOPPe regimen), more frequent use of G-CSF (because of the greater hematological toxicity in the elderly), and renal function.

Smoking has been identified as an RF for the occurrence of BLT by some teams [19-22] but not by others [23-28]. In our series, no woman reported a history of smoking (that might be for cultural reasons), yet smoking is more frequent in the group of BLT+ patients (44.4%) than in others (15.1%), with p = 0.004. In univariate and multivariate analyses, smoking was a statistically significant RF for the occurrence of BLT, with p = 0.006 and 0.038, respectively.

All studies confirmed that the risk of BLT was dose-dependent [29-32]. A cumulative dose of more than 400 mg (i.e., 400 IU) is associated with a high rate of BLT and should be avoided. However, BLT can even occur with cumulative doses below 50 IU [33]. A rapid flow of infusion is also associated with an increased risk of toxicity [34]. Concomitant use of G-CSF with bleomycin has also been associated with the occurrence of BLT [35,36]. In our study, the ABVD regimen (containing 20 IU/m^2^/cycle every 28 days) is an independent RF for the occurrence of BLT compared to the BEACOPPe regimen (containing 10 IU/m^2^/cycle every 21 days), in both univariate (p = 0.03) and multivariate (p = 0.018) analyses, despite the systematic use of G-CSF in BEACOPPe and its occasional use in ABVD.

Kidney failure is also strongly associated with the risk of developing BLT as 80% of bleomycin is eliminated by kidneys [25]. In our study, renal function was normal in most patients, and therefore no significant difference was found between the two groups of patients. Chest radiotherapy was also identified as an RF for the development of BLT [37]. One study showed that patients with HL had a significantly reduced risk of BLT with an interval of more than four weeks between thoracic irradiation and the last dose of bleomycin [37]. In our study, patients who had an indication for radiotherapy developed BLT before irradiation. We were therefore unable to evaluate its impact. Among other RFs described in the literature, bleomycin associated with cisplatin [38] or gemcitabine (in the treatment of germ cell cancers) and use of high-concentration oxygen therapy were reported. The impact of the latter remains controversial [39].

Data on the benefits of PET scan in the early detection of BLT are rare and contradictory. Since BLT leads to diffuse hypermetabolism of the pulmonary parenchyma [40,41], some authors have suggested that intermediate PET scans after two or four cycles might be an effective means of early detection of BLT before the onset of clinical symptomatology and radiological signs [9,42,43].

In our study, a two-cycle PET scan was performed in 11 of the 18 BLT+ patients. It helped us predict the development of BLT in four (36.4%) patients who were asymptomatic at the time of examination and who already had pulmonary hypermetabolism. On the other hand, after four cycles, out of 18 BLT+ patients, only two (12.5%) patients were asymptomatic, although they presented a pulmonary hypermetabolism on the PET scan.

In BLT- patients (n = 106), 85 underwent a PET scan, and four of them had diffuse pulmonary hypermetabolism after four cycles. For these four patients, bleomycin was stopped and the pulmonary hypermetabolism disappeared on the end-of-treatment PET scan, but these patients never showed any clinical or radiological signs of BLT. It was therefore difficult to attribute pulmonary hypermetabolism to BLT.

According to the results of our study, pulmonary hypermetabolism on the intermediate PET scan should be a reason to discuss permanent discontinuation of bleomycin given the risk of BLT and the lack of benefit in patients with complete metabolic remission [15-17]. In addition, based on our study, the intermediate PET scan should not be considered as a reliable tool for the early detection of BLT.

## Conclusions

The findings of this study suggest that ABVD and smoking were RFs for the development of bleomycin lung toxicity. On the other hand, two- and four-cycle PET scans should not be considered as a reliable screening tool for the early detection of this toxicity.
